# Impact of the COVID-19 Pandemic on Seizure Control in Pediatric Epilepsy: Risk Factors and Clinical Outcomes

**DOI:** 10.3390/healthcare13020172

**Published:** 2025-01-16

**Authors:** Jihye Lim, Ja Un Moon

**Affiliations:** 1 Division of Gastroenterology and Hepatology, Department of Internal Medicine, Yeouido St. Mary’s Hospital, College of Medicine, The Catholic University of Korea, Seoul 07345, Republic of Korea; 2 Division of Pediatric Neurology, Department of Pediatrics, Yeouido St. Mary’s Hospital, College of Medicine, The Catholic University of Korea, Seoul 07345, Republic of Korea

**Keywords:** COVID-19, pandemic, epilepsy, children, adolescents, pediatric, seizure

## Abstract

**Background:** Epilepsy is a common neurological disorder in children, associated with significant morbidity and socioeconomic burden. The coronavirus disease 2019 (COVID-19) pandemic disrupted healthcare delivery, potentially exacerbating seizure control among pediatric epilepsy patients. This study aimed to evaluate the pandemic’s impact on seizure characteristics and identify risk factors contributing to seizure exacerbation in children with epilepsy. **Methods:** A retrospective cohort study was conducted using medical records of 84 pediatric epilepsy patients at The Catholic University of Korea Yeouido St. Mary’s Hospital from July 2019 to July 2022. Data were collected on demographics, epilepsy characteristics, and healthcare accessibility. Changes in seizure outcomes were analyzed alongside potential risk factors, including infections and socioeconomic variables. Statistical analyses assessed correlations between these factors and seizure exacerbations. **Results:** Among the 84 pediatric epilepsy patients, 25% experienced significant seizure exacerbations during the COVID-19 pandemic. These included increased seizure frequency (18%), prolonged duration (13%), emergence of new seizure types (4%), and status epilepticus requiring hospitalization (5%). Multivariate analysis identified recent epilepsy diagnosis (<1 year) and low socioeconomic status as independent predictors of seizure worsening (*p* < 0.05). Infections with non-COVID-19 respiratory viruses, such as RSV and influenza, were strongly associated with exacerbated seizure activity (*p* < 0.001). Dissatisfaction with access to epilepsy care further increased the risk of poor seizure control, reflecting the challenges posed by disrupted healthcare systems. Notably, no significant relationship was observed between SARS-CoV-2 infection and seizure outcomes, suggesting that indirect factors, rather than direct viral effects, were primary contributors to seizure exacerbation. **Conclusions:** This study highlights the compounded impact of disrupted healthcare access, socioeconomic challenges, and respiratory viral infections on seizure control during the COVID-19 pandemic. Strategies such as telehealth expansion, regular monitoring, and vaccination against respiratory pathogens are essential to optimize seizure management in future health crises.

## 1. Introduction

Epilepsy is one of the most frequent neurological conditions in children. It is associated with a high economic cost, the development of intellectual disability, psychiatric, and cognitive comorbidities, and a high risk of injury and mortality [1,2,3,4,5].

Although childhood-onset epilepsy is characterized by self-limiting seizures in most cases, about one-third of these children develop anti-seizure medication (ASM)-resistant epilepsy, requiring further optimized advanced, and sophisticated medical therapy.

The World Health Organization (WHO) declared the new coronavirus disease (COVID-19) to be a pandemic on 11 March 2020. The COVID-19 pandemic has affected the health system in the entire world, resulting in significant economic damage [6].

The Centers for Disease Control and Prevention (CDC) suggested that epilepsy, one of the neurological comorbidities, might be a risk factor for COVID-19 infection due to the increased rate of comorbidities associated with these patients. Due to the lack of evidence concerning the associations between epilepsy and susceptibility to COVID-19 infection, this statement has been removed from its website. To the best of our knowledge, children are less frequently affected by COVID-19 infection and are associated with less severe symptoms, lower morbidity, and mortality than adults after COVID-19 infection [6,7,8]. Moreover, acute symptomatic seizures do not seem to occur very often. Complications of COVID-19 infection in children resulting from the severe acute respiratory syndrome coronavirus 2 (SARS-CoV-2) occur in approximately less than 0.5% [9].

However, due to the gradual easing of quarantine restrictions as time goes by, the possibility and susceptibility of COVID-19 infection has increased, which might negatively impact each person, such as patients with epilepsy. Additionally, during the pandemic, not only the SARS-CoV-2 infection itself but changing lifestyle circumstances could also significantly impact patients with epilepsy [10]. Furthermore, Baig et al. suggested that the central nervous system could be a favorable target of SARS-CoV-2 as the angiotensin-converting enzyme-2 (ACE2) receptor, which is highly expressed in brain tissue and host cells, contributes to the source of COVID-19 virus entry points into cells [11,12]. Given that patients with chronic diseases such as epilepsy have overexpressed ACE2 receptors, their susceptibility to COVID-19 infection might be higher, especially under relaxed quarantine restrictions. In addition, experiencing uncontrolled seizures has been highly associated with mortality in epilepsy patients [1] more than those with well-controlled symptoms; therefore, seizure control during the COVID-19 pandemic is crucial. However, a few studies have reported on whether the consequences of the pandemic impact seizure control or not and the outcomes of patients with epilepsy, especially among children. The purpose of this retrospective cohort study was to evaluate the effects of the COVID-19 pandemic on the characteristics of epilepsy and seizure control in pediatric patients with epilepsy during the pandemic. Additionally, the study aimed to examine whether factors such as limited access to epilepsy care, low socioeconomic status, and respiratory viral infections contributed to the exacerbation of seizures in this population during the pandemic.

## 2. Materials and Methods

This retrospective observational study was conducted using the medical records of children and adolescents (aged 0–18 years) diagnosed with epilepsy, who were treated at the pediatric neurology outpatient clinic of The Catholic University of Korea Yeouido St. Mary’s Hospital, a private healthcare institution. The study period spanned from July 2019 to July 2022. The inclusion criteria were as follows: (1) patients aged between 0 and 18 years; (2) patients diagnosed and treated for epilepsy at least 6 months prior to the onset of the COVID-19 pandemic; and (3) patients who had been followed-up for more than 6 months after April 2020, either through in-person outpatient visits or via telemedicine. Exclusion criteria included (1) newly diagnosed patients; (2) patients with video EEG-confirmed psychogenic non-epileptic seizures (PNES); (3) patients with severe psychological disorders (e.g., anxiety, depression); and (4) patients who did not receive follow-up care during the COVID-19 pandemic.

A single pediatric neurologist reviewed the medical charts and extracted data on patient demographics (age, sex, socioeconomic status), age at epilepsy diagnosis, EEG findings, number of anti-seizure medications (ASMs) prescribed, and comorbidities associated with epilepsy. The outcomes related to seizure control were defined according to four key parameters: seizure frequency, seizure duration, occurrence of new seizure types, and the incidence of cluster seizures/status epilepticus. Seizure frequency was recorded as the number of seizures per month and categorized into three groups based on comparison with the frequency observed during the 6 months preceding the pandemic: increase, decrease, or no change. Seizure duration was classified as being more than 50% longer or shorter than the patient’s average duration for that specific type of seizure.

Exposure to SARS-CoV-2 and other respiratory viruses was confirmed via reverse transcription polymerase chain reaction (RT-PCR) analysis of nasopharyngeal and throat swab samples. Factors such as sleep quality, physical activity, and accessibility to epilepsy care during the COVID-19 pandemic were assessed through a custom, non-validated self-reported survey conducted by telephone interview with the caregivers from 2019 to July 2022. Formal verbal informed consent was obtained from all participants prior to the study. Caregivers unable to participate in the survey due to intellectual disabilities or hearing impairments were excluded. All collected data were anonymized, and no patient-identifying information was used at any stage of the study.

The survey consisted of six primary questions: (1) socioeconomic status (income, occupation, education); (2) changes in physical activity during the COVID-19 period; (3) changes in sleep quality during the COVID-19 period; (4) satisfaction with accessibility to epilepsy care (including obtaining ASMs and accessing outpatient clinics/emergency departments); (5) infection with SARS-CoV-2 confirmed by RT-PCR; and (6) infection with other respiratory viruses confirmed by RT-PCR. To improve response rates during the quarantine restrictions, both categorical and binary (yes/no) response formats were employed. Socioeconomic status was categorized into three levels (low, middle, high) based on self-reported income, occupation, and education. Additionally, participants were asked about their sleep quality, including sleep duration, presence of sleep disturbances, and adherence to sleep routines.

Seizure outcomes related to SARS-CoV-2 and other respiratory viruses were monitored throughout the follow-up period, not limited to periods of active infection. Each patient was followed regularly with EEG assessments every 6 months for a period of at least two years between July 2019 and July 2022. EEG findings were categorized as “worsened” if there was any significant increase in discharge, emergence of new foci, worsening background activity, or global slowing. Conversely, EEG findings were categorized as “improved” if there was a reduction or resolution in epileptiform patterns, background abnormalities, or focal/generalized slowing.

The classification of epilepsy etiology and seizure types followed the guidelines proposed by the International League Against Epilepsy (ILAE) in 2014 and 2017, respectively [13,14]. Drug-resistant epilepsy (DRE) was defined as the presence of seizures in the preceding 12 months despite treatment with two or more ASMs [15].

Statistical analysis was performed using descriptive statistics, with categorical variables reported as frequencies and percentages, and continuous variables reported as means ± standard deviations. Comparisons of continuous variables were made using the *t*-test or Mann–Whitney U test, while categorical variables were analyzed using chi-square or Fisher’s exact tests. Univariate logistic regression was conducted to identify potential risk factors for worsening seizures during the COVID-19 pandemic. A *p*-value of <0.05 was considered statistically significant. All statistical analyses were performed using SPSS software (version 26.0; IBM Corp., Armonk, NY, USA) and R software (version 3.6.0; https://www.r-project.org). This study was approved by the Institutional Review Board of The Catholic University of Korea (SC23RISI0028). Due to the anonymized nature of the questionnaire-based dataset, the requirement for formal ethical review and informed consent was waived for the study.

## 3. Results

### 3.1. Demographic and Clinical Characteristics of Children with Epilepsy Before COVID-19 Pandemic

**Figure 1:**
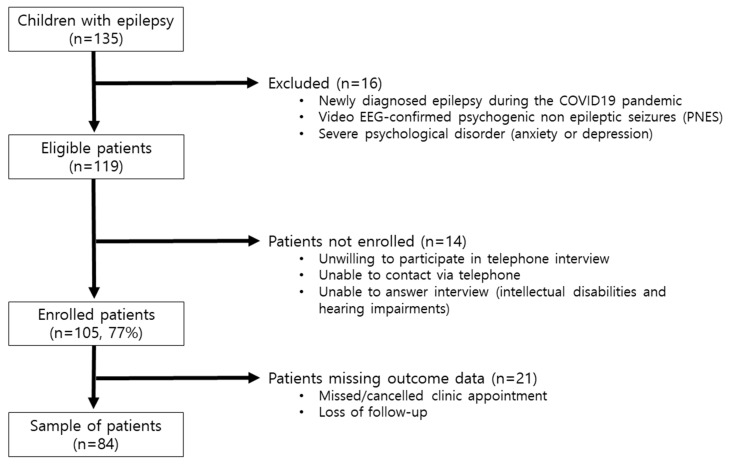
Flowchart of patient enrollment in the study.

A total of 135 pediatric patients with epilepsy were initially recruited during the COVID-19 pandemic period. Of these, 84 patients (38 girls and 46 boys) were ultimately included in the study after exclusions (Figure 1). Fourteen patients were excluded for being unable to provide verbal consent, and twenty-one patients were excluded due to lack of follow-up during the study period. The demographic and clinical characteristics of the patients prior to the COVID-19 pandemic are summarized in Table 1.

The mean age of the 84 patients was 10.8 years (range, 2–15 years), with the mean age at seizure onset being 3.5 years (range, 1 month–13 years). A quarter of the patients had caregivers with a middle or high socioeconomic status, while 20% were living in self-reported low socioeconomic conditions. A total of 30% of the patients had generalized onset seizures, 14% had focal onset seizures, and the remaining 56% had unknown or unclassified seizure types. In terms of etiology, more than half of the patients (56%) had an unknown underlying cause for their epilepsy, followed by genetic etiologies (27%) and structural causes (12%).

Before the COVID-19 pandemic, 77% of patients were treated with anti-seizure medication (ASM) monotherapy. The most commonly prescribed ASMs included levetiracetam, valproic acid, lamotrigine, clonazepam, topiramate, and oxcarbazepine. The dosages varied among patients, but a standard starting dose was used for each medication. For example, the starting dose of levetiracetam was 30 mg/kg, valproic acid 25 mg/kg, lamotrigine 2 mg/kg, clonazepam 0.1 mg/kg, topiramate 5 mg/kg, and oxcarbazepine 10 mg/kg per day. Among the patients, eight were classified as having drug-resistant epilepsy (DRE), although none required hospitalization during the study period. The majority of patients (approximately two-thirds) had no comorbidities, followed by those with neuropsychiatric disorders such as attention deficit hyperactivity disorder (ADHD) and autism spectrum disorder (13%), and those with developmental delay or intellectual disability (8%). Developmental delay was assessed using the Bayley Scales, which evaluates cognitive, language, motor, and social delays in children under six years of age. Intellectual disability was defined as an intelligence quotient (IQ) of 70 or below, accompanied by adaptive functioning deficits.

### 3.2. Seizure Changes During the COVID-19 Pandemic Era

In this study, seizure worsening was defined as an increase in the frequency or duration of seizures, the onset of new seizure types or clusters, or the occurrence of status epilepticus during the COVID-19 pandemic period. A total of 25% of patients experienced significant deterioration in seizure control. This included an increase in seizure frequency in 18% of patients, prolonged seizure duration in 13%, the emergence of new seizure types in 4%, and the occurrence of prolonged seizures in 5%. Notably, 15% of patients exhibited all of these changes simultaneously. Status epilepticus was reported in four patients, all of whom required hospitalization (Table 2).

EEG data showed that, for approximately 50% of patients, there was either no change or an improvement compared to pre-pandemic recordings. However, 8% of patients experienced worsening EEG findings, including an increase in spike frequencies and the emergence of new seizure foci. Additionally, 42% of patients had missing or incomplete EEG data due to the cancellation of appointments during the pandemic.

### 3.3. Risk Factors of Significantly Worsening Seizures During the COVID-19 Pandemic

Twenty-one (25%) of the 84 patients experienced a significant worsening of seizures during the pandemic (Table 3). Univariate analysis was performed to identify factors that might influence the likelihood of seizure exacerbation, including variables such as “mean age”, “mean age at seizure onset”, “socioeconomic status”, “time since epilepsy diagnosis”, “sleep cycle”, “physical activity”, “COVID-19 infection”, “respiratory virus infection”, and “accessibility to epilepsy care” (Table 3).

A shorter duration of epilepsy diagnosis (less than one year) was significantly associated with an increased likelihood of seizure worsening (*p* = 0.009, 95% CI: 0.04–0.64). In addition, patients from lower socioeconomic backgrounds were more likely to experience seizure exacerbation (*p* = 0.023, 95% CI: 1.19–11.41). Although 25% of patients were exposed to SARS-CoV-2 during the pandemic, no significant association was found between SARS-CoV-2 infection and worsening seizures (*p* = 0.88). Interestingly, infections with other respiratory viruses, including respiratory syncytial virus (RSV) and influenza A and B viruses, were significantly associated with an increased risk of seizure exacerbation (*p* < 0.001, 95% CI: 3.14–35.94). Furthermore, patients who reported dissatisfaction with their accessibility to epilepsy care were significantly more likely to experience worsening seizures (*p* = 0.001).

## 4. Discussion

The global COVID-19 pandemic has had a profound impact across various fields, including those related to epilepsy. Given the extended duration and escalating risks associated with the pandemic, it is crucial to explore the factors that contribute to the exacerbation of seizures in pediatric patients with epilepsy during this period.

The present study aimed to assess the impact of the COVID-19 pandemic on pediatric patients with epilepsy and to identify the risk factors associated with seizure exacerbation. Notably, 25% of patients in our cohort experienced a worsening of seizure frequency, duration, or both, while 75% reported stable seizure control. This finding is consistent with the results of Momani et al., who reported that 22.7% of children under the age of 16 experienced worsened seizure control during the 2021 COVID-19 lockdown in Jordan [16]. Comparable findings have been observed not only in pediatric populations but also in adult patients. For instance, studies conducted in Saudi Arabia and Spain revealed that approximately 27% and 29.5% of adult epilepsy patients, respectively, had an increase in seizure frequency during the pandemic, reinforcing the global nature of this issue [17,18]. This is further supported by a study in Brazil, which found that one-quarter of patients experienced increased seizure frequency during the 2021 pandemic [19].

Most of our findings regarding worsened seizures were in alignment with results from other studies that utilized similar survey methodologies [18,20,21,22,23]. According to our data, children from lower socioeconomic backgrounds and those dissatisfied with their access to epilepsy care were at an increased risk of seizure exacerbation during the pandemic.

Other risk factors included a recent epilepsy diagnosis (less than a year), and infection with respiratory viruses like RSV and influenza A and B, which have not been previously identified in the literature as a risk factor for seizure exacerbation. Interestingly, SARS-CoV-2 infection was not identified as a direct risk factor for seizure exacerbation, aligning with other studies that have failed to link COVID-19 infection with the onset of seizures in individuals with epilepsy. This contrasts with the Severe Acute Respiratory Syndrome (SARS) virus, which was found to exacerbate seizures during the previous outbreaks [20,24]. Additionally, the greater impact of the COVID-19 pandemic on patients with epilepsy, as compared to the SARS outbreak, could explain the observed differences [25].

Our study identified that children who were dissatisfied with their accessibility to epilepsy care were at a significantly higher risk of experiencing worsened seizures. As previously mentioned, limited access to healthcare services for children with epilepsy could exacerbate their neurological condition and increase the likelihood of complications associated with pre-existing conditions. Notably, the pandemic led to significant disruptions in epilepsy care, with many patients experiencing reduced access to essential diagnostics, such as EEGs, further complicating the management of seizure control. Of the 25% of patients who experienced worsening seizures, only 8% demonstrated worsened EEG results, while 42% of the patients had EEGs either not performed or canceled due to the mass quarantine measures. Our findings support prior surveys indicating that access to EEG evaluations was dramatically reduced during the pandemic, with some studies reporting a 75–90% reduction in outpatient and inpatient EEG evaluations [25,26]. Panda et al. identified a lack of transportation as a barrier to accessing healthcare facilities, which hindered the availability of medications, including ASMs [27]. For patients with chronic conditions such as epilepsy, particularly in regions with restricted healthcare services, the COVID-19 pandemic may have exacerbated their underlying conditions [27,28]. Interestingly, a substantial portion of children in our study (two-thirds) reported satisfaction with their access to epilepsy care, despite the challenges posed by the pandemic. This could be explained by the rapid transition to telehealth services in South Korea, which provided an alternative means of accessing care. A previous Italian study on the transition to telehealth during the pandemic found that most respondents considered remote consultations to be more beneficial than traditional healthcare services [28]. Previous research also corroborates the observation that South Korea’s expedited adoption of telehealth services during the pandemic significantly enhanced patient satisfaction. This improvement can be attributed primarily to the reduction in burdens related to long-distance travel and the prevention of work absenteeism for both patients and their caregivers, which ultimately facilitated greater accessibility and efficiency in healthcare delivery [29]. However, it is important to note that not all children in our cohort had access to these services, and for some, the lack of in-person consultations and EEG evaluations may have contributed to suboptimal treatment and worsening of seizures. Thus, in the context of limited accessibility to epilepsy care, the establishment of widely accessible telehealth programs could help maintain continuity of care, ensuring access to physicians, anti-seizure medications (ASMs), information, neurologic evaluations, and the identification of mental health concerns.

As patients with epilepsy are vulnerable to dramatic changes in their socioeconomic situation [30], lower socioeconomic status may further impair seizure control [18,22,31,32]. Previous research has demonstrated that lower socioeconomic status is associated with a higher prevalence of epilepsy and poorer seizure control [33]. Individuals with lower educational attainment, those who are unemployed, or those experiencing financial hardship may face significant obstacles in accessing necessary healthcare [22,32,34,35]. These obstacles—such as difficulties affording medications, contacting healthcare providers, or limited access to telemedicine—can have a direct impact on seizure control. Consequently, when evaluating the risk of seizure exacerbation, it is essential to consider both access to epilepsy care and socioeconomic factors in tandem. Another critical factor potentially linking socioeconomic status to seizure exacerbation is psychological stress [36,37]. Psychological stress, which has been extensively documented during the pandemic, is another important factor influencing seizure control. Socioeconomic hardships arising from the quarantine were identified as one of the major stressors affecting patients with epilepsy during the pandemic. Furthermore, the emotional distress caused by social isolation, concerns about health, and disruptions to daily life may have contributed to the exacerbation of seizures [18,22,23,38,39,40]. Previous studies have shown that patients with epilepsy are more vulnerable to psychological stress, which is known to have a direct impact on seizure frequency [18,41]. Moreover, it is well established that psychological stress can exacerbate epileptic activity [2,42]. Sánchez-Larsen et al. emphasized that emotional distress is a critical factor in seizure control, as it plays a pivotal role in the failure to manage seizures [2,42]. Although our study did not identify a significant association between psychological stress and worsening seizure activity—primarily due to the absence of stress-related questionnaires—it remains reasonable to hypothesize that the combined effects of socioeconomic and psychological stressors during the lockdown period may have increased the risk of seizure exacerbation for many children with epilepsy. This underscores the need for further investigation into the multifaceted factors influencing seizure control and the potential role of psychological stress within this context.

In addition to psychological and socioeconomic factors, other changes in lifestyle, such as altered sleep patterns and reduced physical activity, were also identified as potential contributors to seizure exacerbation during the pandemic [43,44]. However, our findings did not support a direct correlation between these lifestyle changes and worsening seizure control. This discrepancy may be attributed to differences in patient populations, healthcare systems, or the timing of data collection. Some studies have suggested that the increased time spent at home during lockdown periods may have led to improved adherence to ASMs [38,45] and better sleep schedules, which could result in better seizure control [9]. This contrast underscores the need for further research to better understand the impact of lifestyle changes during the pandemic on seizure outcomes.

A key risk factor identified in this study for worsening seizures was a recent epilepsy diagnosis (less than one year). This subgroup of children is particularly vulnerable to seizure fluctuations, as the condition may not yet be stable, and factors such as improper ASM dosage, poor medication adherence, and difficulties in the adjustment of treatment can contribute to poor seizure control. Additionally, the absence of EEG monitoring, due to logistical challenges during the pandemic, exacerbated these issues, making it difficult to assess the effectiveness of treatment or make necessary adjustments. Previous literature, irrespective of ethical considerations or national income [21], has shown that difficulties in accessing EEGs, obtaining medications, and consulting clinicians were significant challenges for families during the pandemic, which are significant factors in worsening seizure control [20,21,24,38]. Although telemedicine emerged as an alternative during the pandemic to address the challenges of in-person visits [46,47,48], the limited access to outpatient and inpatient EEGs persisted. As such, clinical decision making without EEG data may result in suboptimal treatment. These findings highlight the importance of adjusting ASM dosages and monitoring treatment response with EEGs, particularly in children recently diagnosed with epilepsy (less than a year).

Another interesting and somewhat unexpected finding in our study was the association between respiratory viral infections, such as RSV and influenza A and B, and worsening seizure control. Although one-third of patients in our study were infected with SARS-CoV-2, no seizures were reported to coincide with COVID-19 infection [49,50,51]. This aligns with findings from other studies suggesting that COVID-19 itself does not appear to directly cause seizures [8,52].

In contrast to SARS-CoV-2, infections with respiratory viruses such as RSV and influenza were identified as potential risk factors for seizure exacerbation. This may reflect the extended lockdown period, which led to an increase in infections from seasonal respiratory viruses. During the first year of the pandemic, non-pharmaceutical interventions (NPIs) such as social distancing, school closures, restrictions, hand sanitation, and mask mandates significantly reduced the spread of respiratory viruses [53,54]. In other words, the same restrictions implemented to control the spread of SARS-CoV-2 also led to the suppression of seasonal respiratory viruses, which were significantly reduced during the early phases of the COVID-19 pandemic [55,56]. However, as quarantine restrictions were gradually relaxed following the first wave of COVID-19, infections caused by the transmission of seasonal respiratory viruses via the respiratory route notably increased in late 2020 and early 2021 [57,58]. Of particular relevance to transmission dynamics, childcare and daycare centers were largely reopened and remained operational during these periods of restriction. The generally poor health conditions of children infected with these viruses—characterized primarily by fever, fatigue, and poor oral intake—may act as predisposing factors that exacerbate the occurrence of seizures. Viral infections, in conjunction with malnutrition, can impair immune function, potentially leading to uncontrolled seizures [1,59].

Given that one of the respiratory viruses detected in our study was RSV, the possibility of hypoxic insults resulting from respiratory distress secondary to RSV bronchiolitis may serve as a risk factor for worsening seizures in children with epilepsy. Previous studies have suggested that RSV-induced neurological complications could be due to direct invasion of the CNS by RSV, which was later confirmed by the elevated presence of several cytokines and RSV RNA in cerebrospinal fluid analysis [55,57,58,60,61,62]. Similarly, it has been demonstrated that an increased level of cytokines during influenza infection contributes to neurological complications [54,55,61,62].

Our study aligns with previous findings indicating that the prevalence of worsening seizure control in patients with epilepsy during the pandemic varied significantly. Earlier studies reported that the prevalence of worsening seizures in patients with epilepsy was generally below 10%, or showed no significant changes, whereas recent studies have found a higher prevalence (12–28%) [23,28,38,45]. Several factors could explain these discrepancies. First, differences in sample size and age groups may contribute to these variations. Unlike recent studies that included a larger cohort of younger patients, previous studies primarily focused on older age groups. Additionally, many of the earlier studies were conducted before the winter of 2020, a period characterized by minimal outbreaks of respiratory viruses other than SARS-CoV-2 [55]. This timing discrepancy might account for the observed differences in neurological complications related to respiratory viral infections in children with epilepsy. Another potential explanation lies in the differing clinical presentations of SARS-CoV-2 and other respiratory viruses. COVID-19 in children is often asymptomatic or presents with mild symptoms, leading to undetected or underestimated outcomes. In contrast, infections caused by other respiratory viruses typically present with more overt symptoms, such as fever, fatigue, myalgia, and poor oral intake, all of which are known to predispose children to acute seizures. This is consistent with our findings, where five patients, all infected with influenza A virus rather than COVID-19, exhibited seizures accompanied by fever.

By mid-2022, quarantine restrictions were relaxed to varying degrees, either fully or partially, leading to delayed and unpredictable outbreaks of various respiratory viruses [55]. This increase in viral transmission has likely contributed to a rise in infections worldwide, further complicating the management of children with epilepsy. Therefore, pediatric neurologists must not only provide appropriate epilepsy care but also adapt their clinical practices to address the evolving landscape of respiratory viruses, aiming to achieve better seizure control.

Our study presents several strengths, including a thorough analysis of pediatric patients across different age groups during an extended lockdown period, as well as the inclusion of testing for a wide array of respiratory viruses, including SARS-CoV-2. However, there are notable limitations that should be considered. First, although our sample size is sufficiently powered to detect odds ratios greater than 1.5, it may not be large enough to identify smaller, yet clinically significant effects. Therefore, the findings should be viewed as preliminary. Additionally, due to the small sample size, the associations between short-term epilepsy diagnosis (less than one year), low socioeconomic status, and the risk of exacerbated seizures in children with other respiratory infections in South Korea may not be broadly applicable.

Second, the retrospective design, based on medical chart reviews, introduces potential biases, particularly in the interpretation of EEG outcomes, which can be influenced by the timing of seizures. Nonetheless, regular longitudinal EEG assessments provided a more comprehensive understanding of seizure outcomes over time, thus helping mitigate recall bias. To minimize bias further, objective clinical data—such as seizure frequency, EEG findings, and medication use—were used, thereby reducing information bias and ensuring more reliable measures of seizure control.

Third, the data were collected from a single academic center, which limits the generalizability of our findings to the wider pediatric population in South Korea and may not extend to other countries. Fourth, the reliance on self-reported interviews introduces potential recall biases. To minimize recall bias, structured, binary (yes/no) and categorical response formats were used to simplify responses and enhance accuracy. Telephone interviews allowed for clarification of questions, ensuring better understanding and reducing recall errors. Additionally, regular EEG assessments every 6 months provided objective longitudinal data, reducing reliance on patient/caregiver memory for seizure outcomes. Finally, the use of a bespoke, non-validated questionnaire instead of standardized tools to assess factors contributing to seizure exacerbation constrains the depth and reliability of the analysis.

Given these limitations, larger, well-designed studies are needed to build on these findings and provide a clearer understanding of how seizures are affected during pandemics. Future research should also include mental health assessments to better explore the relationship between COVID-19, respiratory infections, and seizure outcomes in children.

## 5. Conclusions

In conclusion, this study highlights two previously underreported factors contributing to the exacerbation of seizures in pediatric epilepsy patients during the COVID-19 pandemic: a recent diagnosis of epilepsy (within the past year) and the exacerbating effects of respiratory viral infections, including influenza and RSV. The findings underscore the importance of precise adjustment of ASM dosages, regular follow-up, and periodic EEG monitoring, particularly for children newly diagnosed with epilepsy. Furthermore, our results suggest that respiratory viral infections, including but not limited to SARS-CoV-2, significantly impacted seizure control. These findings emphasize the need for integrated management strategies, including vaccination, enhanced hygiene practices, and the expansion of telehealth services, to mitigate the risk of seizure exacerbation. Continuous surveillance of respiratory virus epidemiology, coupled with proactive monitoring and intervention, is critical to optimizing seizure management in children with epilepsy, particularly during health crises such as the ongoing pandemic.

## Figures and Tables

**Table 1:** Demographic and clinical characteristics of subjects before the COVID-19 pandemic.

Characteristics	* Values
**Total patient**	84 (100)
Female	38 (45)
Male	46 (55)
**Mean age** (years ± SD)	10.8 (±4.6)
**Mean age at seizure onset** (years ± SD)	3.5 (±2.6)
**Socioeconomic state ^a^**	
Middle/High	67 (80)
Low	17 (20)
**Time from epilepsy diagnosis**	
Less than 1 year	37 (45)
1–3 years	17 (20)
More than 3 years	30 (35)
**Seizure classification**	
Focal onset	12 (14)
Generalized onset	58 (30)
Unclassified/Unknown onset	14 (56)
**Etiologies of epilepsy**	
Genetic ^b^	23 (27)
Structural	10 (12)
Metabolic	1 (1)
Immune	2 (3)
Infectious	1 (1)
Unknown	47 (56)
**Number of anti-seizure medications**	
Monotherapy	65 (77)
Polytherapy	19 (23)
**Drug-resistant epilepsy**	8 (9.5)
**Comorbidities associated with epilepsy**	
Developmental delay/Intellectual disability	7 (8)
Neuromuscular disease	2 (2)
Neuropsychiatric disorders	11 (13)
Cerebral palsy	4 (5)
Cerebrovascular disease	2 (2)
Genetic disorders	3 (2)
None/Unclassified	55 (68)

* Values are presented as mean ± standard deviation or number (%). SD, standard deviation; ^a^ subject socioeconomic status considering income, occupation, and education. ^b^ genetic generalized epilepsy (previously known as idiopathic generalized epilepsy) was considered to be of genetic etiology.

**Table 2:** Seizure profile during the COVID-19 pandemic.

Characteristics	Values N (%)	Characteristics	Values N (%)
**Seizure worsening ^a^**		**Occurrence of new seizures**	
Yes	21 (25)	Yes	3 (4)
No	63 (75)	No	81 (96)
**Seizure frequency**		**Occurrence of status epilepticus**	
Increased	15 (18)	Yes	4 (5)
No change	48 (57)	No	80 (95)
Decreased/No seizure	21 (25)		
**Seizure duration ^b^**		**Electroencephalography**	
Longer	11 (13)	Unchanged/Improved	42 (50)
No change	62 (74)	Worsened ^c^	7 (8)
Shorter	11 (13)	Missing data/Undone	35 (42)

Values are presented as numbers (%). ^a^ Defined as an increase in the frequency or duration of seizures, the occurrence of new seizures, and seizure clusters/status epilepticus during the COVID-19 pandemic. ^b^ Defined by the duration of seizures more than 50% longer/shorter than the average duration of seizures in the same patient with the same type of seizure. ^c^ Defined by any significant increase in epileptiform discharges and the emergence of second or third focus compared to the previous study.

**Table 3:** Univariate analysis of risk factors aggravating seizures during the COVID-19 pandemic seizure profile during the COVID-19 pandemic.

Characteristics	Worsening Seizure	Without Worsening Seizure	*p* Value	OR (95% CI)
**Total patients**	21	63		
**Mean age** (years ± SD)	8.8 (±2.1)	9.1 (±1.3)	0.532	0.91 (0.69–1.21)
**Mean age at seizure onset** (years ± SD)	1.8 (±1.2)	2.2 (±2.4)	0.461	0.9 (0.69–1.18)
**Socioeconomic state ^a^**			0.023	3.69 (1.19–11.41)
High	13 (62)	54 (86)		
Low	8 (38)	9 (14)		
**Time from epilepsy diagnosis**			0.009	0.16 (0.04–0.64)
Less than 1 year	15 (72)	22 (35)		
1–3 years	3 (14)	14 (22)		
More than 3 years	3 (14)	27 (43)		
**Number of antiseizure medications**			0.652	0.75 (0.22–2.59)
Monotherapy	17 (81)	48 (76)		
Polytherapy	4 (19)	15 (24)		
**Sleep cycle**			0.556	0.69 (0.29–2.09)
Worse	4 (19)	16 (25)		
Better/Not affected	17 (81)	47 (75)		
**Physical activity**			0.614	0.77 (0.29–2.09)
Worse	9 (43)	31 (49)		
Better/Not affected	12 (57)	32 (51)		
**Infection with SARS-CoV-2**			0.88	0.91 (0.28–2.94)
Yes	16 (76)	49 (78)		
No	5 (24)	14 (22)		
**Infection with other viruses ^b^**			<0.001	10.62 (3.14–35.94)
Yes	17 (80)	18 (29)		
No	4 (20)	45 (71)		
**Accessibility to epilepsy care ^c^**			0.001	0.14 (0.04–0.47)
Satisfied	12 (57)	57 (90)		
Not satisfied	9 (43)	6 (10)		

Values are presented as numbers (%). SARS-CoV-2, severe acute respiratory syndrome coronavirus 2; OR, odds ratio; CI, confidence interval; ^a^ Subject socioeconomic status considering income, occupation, and education. ^b^ Included respiratory syncytial virus, influenza A and B. ^c^ Included obtaining anti-seizure medication and accessing healthcare facilities, such as outpatient care and emergency departments.

## Data Availability

The data presented in this study are available from the corresponding author upon reasonable request.
